# “If There Are Restrictions Within the Restrictions, That's When You Can Probably Get Concerned”: Key Indicators for Untying Vegetarianism and Veganism From Eating Disorder Pathology

**DOI:** 10.1002/eat.24475

**Published:** 2025-05-23

**Authors:** Courtney P. McLean, Kathleen de Boer, Megan Bray

**Affiliations:** ^1^ Department of Psychology, Counselling, and Therapy La Trobe University Melbourne Victoria Australia; ^2^ School of Psychological Science Monash University Melbourne Victoria Australia; ^3^ Department of Psychological Sciences Swinburne University Melbourne Victoria Australia; ^4^ School of Human Movement and Nutrition Sciences University of Queensland St Lucia Australia

**Keywords:** dietary adherence, eating disorders, experiences, pathological eating, qualitative, vegan, vegetarian

## Abstract

**Objective:**

Changes in eating patterns and/or food exclusion strategies, including the uptake of vegetarianism and veganism, may reflect disordered behaviors and attitudes in people with eating disorders. For this reason, health professionals often attempt to assess whether a client's vegetarianism or veganism is tied to, or driven by, their eating disorder. Yet this may be difficult considering a lack of formally recognized guidelines for the treatment of vegetarians and vegans with an eating disorder, meaning that often a one‐size‐fits‐all approach to treating these groups is employed. This study aimed to integrate lived eating disorder perspectives to qualitatively inform indicators of potential pathological vegetarian or vegan adherence in people with an eating disorder.

**Method:**

Seventeen participants (aged 19–48, 76% [*n* = 13] female, 47.06% [*n* = 8] vegetarian) with a history of receiving eating disorder treatment were recruited.

**Results:**

Five themes were identified: (1) *Timing matters*, (2) *Explore motivations for dietary adherence*, (3) *Fear reaction causes for concern*, (4) *Flexibility within vegetarianism or veganism*, and (5) *Hold space for eating disorder deception.*

**Discussion:**

Our findings demonstrate several key indicators that may be useful areas of discussion in clinical practice when working with vegetarian and vegan clients. Being able to potentially quantify genuine vegetarian or vegan adherence from eating disorder‐driven behaviors and attitudes provides a valuable stepping stone to the future development of clinical guidelines for the treatment of people adhering to these dietary groups.

**Summary**

- This study sought to categorize key indicators to ascertain potential genuine vs. disordered vegetarianism or veganism in people with an eating disorder (i.e., whether a client's dietary adherence is or is not an expression of their eating disorder diagnosis). We found that the timing of the onset of dietary adherence, motivations for adherence, fear reaction to meat and/or animal products, and flexibility in eating were important areas for further exploration in clinical practice.

## 1 Untying Vegetarian and Vegan Adherence From Eating Disorders: Key Indicators for Querying in Clinical Practice

Changes in eating patterns and/or food exclusion strategies, such as the restriction of meat and/or animal products, may reflect disordered behaviors and attitudes in people with eating disorders. For this reason, vegetarianism and veganism have at times been associated with eating psychopathology within the field; deemed a socially acceptable method of restricting food choices and groups (Kadambari et al. 1986; Timko et al. 2012). For example, estimates of the prevalence of vegetarianism in people with an eating disorder have ranged between 42% and 54% (Heiss et al. 2021; Kadambari et al. 1986; O'Connor et al. 1987), relative to 4%–12% in the general population (Mensink et al. 2016; Morgan 2019; Vergeer et al. 2020). However, the exact causal relationship between vegetarian and vegan diets and eating disorder symptomology remains unclear, with much of the existing literature being cross sectional (McLean et al. 2022a).

Health professionals typically work with clients to become less rigid in their dietary attitudes and may also include working toward the reconsumption of meat and/or animal products (McMaster et al. 2020). However, recent literature exploring the perceptions of vegetarians and vegans found the consumption of meat and/or animal products as part of treatment may not necessarily be beneficial to recovery (McLean et al. 2025). Lived experience voices likened the reconsumption of meat and/or animal products to being faced with an ultimatum, whereby they felt like they needed to choose between maintaining their dietary status and receiving treatment. This may be particularly consequential in individuals whose dietary adherence to vegetarianism or veganism is not an expression of their eating disorder diagnosis (e.g., “genuine” vegetarian or vegan). In this group of individuals, the required consumption of meat and/or animal products was noted to have implications for higher treatment dropout rates and reduced feelings of autonomy (McLean et al. 2025).

When working with vegetarian or vegan clients, health professionals may attempt to assess whether their vegetarianism or veganism is tied to or driven by their eating disorder, and to what extent. However, this brings many challenges. Eating disorders are frequently associated with denial and the concealing of eating behaviors (Howard et al. 2023), resulting in low or delayed help‐seeking and challenges in symptom assessment (Ali et al. 2020). Secondly, there are no formally recognized guidelines for the treatment of vegetarians and vegans with an eating disorder, meaning that there may be inconsistent treatment approaches across healthcare providers. This may be particularly relevant for how health professionals may assess how their client's vegetarianism or veganism may be serving them and their eating disorder. Being able to quantify genuine vegetarian or vegan adherence from eating disorder‐driven behaviors and attitudes may be useful in clinical practice. The recently developed Vegetarian Vegan Eating Disorder Screener (V‐EDS) is a promising tool designed to uniquely screen eating disorder symptoms in people following vegetarianism or veganism (McLean et al. 2024a, 2024b). However, having indicators that may be integrated into clinical practice would allow for modified treatment processes for individuals whose vegetarianism or veganism is not deemed to be driven by their eating disorder. This may assist clients in maintaining autonomy and treatment adherence (McLean et al. 2025). Using lived experience perspectives, this study aims to identify potential indicators of pathological vegetarian or vegan adherence in people with an eating disorder.

## 2 Methods

### 2.1 Study Design and Methodology

This study obtained ethics approval from La Trobe University Human Research Ethics Committee. The COREQ guidelines were followed for the reporting of this study (see Supporting Information; (Tong et al. 2007)). A qualitative phenomenological research design was used to capture participant perspectives on the potential indicators that may inform pathological vegetarianism or veganism (Sundler et al. 2019). This approach is most suited to the present study as it provides a useful method of exploring the lived experiences of our participants and acknowledges the unique personal viewpoint (Clancy 2013; Wilson 2015).

The research team consisted of three members. The first author is an early career researcher in eating disorders and vegetarianism/veganism, the second author is an early career researcher and eating disorder credentialed clinical psychologist, and the third author is a PhD candidate and dietitian in eating disorders. Dietary adherence ranged from vegan, pescatarian, to omnivorous, with each author contributing unique perspectives to the study. There was also diversity among the team in terms of professional experience (e.g., researchers, health professionals [dietitian, clinical psychologist]) which added to the richness of the analytical process. To address the influence of biases in the study, self‐reflexive methods were integrated into analysis processes, including maintaining reflexive journals and regular discussion of coding processes between authors. Data from this study were drawn from a larger project exploring the perceptions of eating disorder treatment as a vegetarian or vegan (McLean et al. 2025).

### 2.2 Recruitment Procedures

Participants were purposively recruited through national and statewide eating disorder organizations and professional networks of the research team. The inclusion criteria were (a) 18 years or over, (b) living in Australia, and (c) having previously sought eating disorder treatment while adhering to a vegetarian or vegan diet. Treatment could include any formal psychological or dietetic services targeted toward eating disorder treatment, with no restrictions on the treatment duration. We adopted a broad definition of vegetarianism to capture the full spectrum of plant‐based dietary patterns and to reflect the real‐world variability in how people interpret and adopt vegetarianism during treatment. Seventeen participants consented to participate.

### 2.3 Data Collection

Participants responded to an expression of interest survey which included the explanatory statement and consent form, demographic questions, and questions relating to their eating disorder history. Those who met the inclusion criteria were invited to an interview, and a copy of the Explanatory Statement and interview guide was provided. Participants met with CM using video conferencing software, Zoom. First, participants were re‐informed of the purpose and aim of the research, and elements of consent were verbally reviewed. The interview guide was used throughout the interview and integrated open‐ended and probing questions (see McLean et al. (2025) for the interview guide). The interviews took an average of 31.28 min (SD = 7.68) and were conducted between June and August 2024. Recruitment continued until data saturation was reached, which provided a sufficient sample size to explore this novel area of research across a range of demographic characteristics (e.g., dietary adherence, eating disorder diagnosis, treatment type; (Braun and Clarke 2021a; Vasileiou et al. 2018)). The researcher had no relationship with any participants prior to study commencement. No repeat interviews were conducted.

### 2.4 Data Analysis

Interview recordings were transcribed using Otter.ai, then reviewed for accuracy and anonymized by the research team. All participants were provided a two‐week opportunity to review their transcript and amend any inaccuracies. Final transcripts were independently coded by researchers CM and KDB using reflexive thematic analysis guided by Braun and Clarke (Braun et al. 2022; Braun and Clarke 2021b). Analysis followed inductive reasoning (i.e., data‐driven), where data were coded based on the authors' understanding of codes and themes, rather than supporting an existing theoretical framework (Thomas 2006). Using NVivo, coders first conducted two practice interviews, which involved independently coding the interviews, then meeting to discuss processes. The remaining transcripts were evenly divided between CM and KDB. Coders deeply *familiarized* themselves with the data by reading the transcripts. In the second stage, *initial coding*, an open coding approach to label segments of text within the dataset was used. As our coding process involved multiple coders, we employed a collaborative and reflexive approach, aiming to achieve rich interpretations of meaning rather than attempting to achieve consensus (Byrne 2022). The *generating themes* stage involved coders collaboratively grouping codes into categories to generate main themes, which were then *reviewed* by MB, who provided feedback and impressions on possible meanings. This led to stage five, *theme finalization*, and stage six, *interpreting and reporting* of the code labels, codes, and themes during the write‐up phase.

## 3 Results

### 3.1 Participant Characteristics

Seventeen participants took part in the interviews and had an average age of 27 years (SD = 6.94, range = 19–48). Most participants identified as female (76.47%, *n* = 13) and were vegetarian (47.06%, *n* = 8) with varying eating disorder diagnoses at the time of seeking treatment. See Table 1 for full participant characteristics. Changes in dietary adherence throughout treatment, including at the time of the interview, were not systematically collected.

**TABLE 1:** Participant demographic characteristics.

Characteristic	Subcategory	*n* (%)
Gender	Female	13 (76.47)
	Nonbinary	3 (17.65)
	Prefer not to disclose	1 (5.88)
State of residence*	New South Wales	4 (23.53)
	Victoria	8 (47.06)
	Queensland	2 (11.76)
	Western Australia	1 (5.88)
	Tasmania	2 (11.76)
Eating disorder diagnosis at time of adhering to vegetarian or vegan diet*	Anorexia nervosa	6 (35.29)
	Atypical anorexia nervosa	4 (23.53)
	Binge eating disorder	1 (5.88)
	Bulimia nervosa	2 (11.76)
	EDNOS	1 (5.88)
	Multiple diagnoses	3 (17.65)
Eating disorder duration	1–5 years	7 (41.18)
	6–10 years	4 (23.53)
	11–15 years	4 (23.53)
	16–20 years	0 (0.0)
	More than 20 years	2 (11.76)
Received inpatient care	Yes	8 (47.06)
	No	9 (52.94)
Vegetarian/vegan status	Vegetarian	8 (47.06)
	Semivegetarian^	1 (5.88)
	Pescatarian >	1 (5.88)
	Vegan	7 (41.18)

*Note*: *Rows in subcategory may not add up to 100 due to rounding. ^ Refers to individuals who primarily follow a vegetarian diet but occasionally consume meat. > Refers to individuals who exclude meat but consume fish.

### 3.2 Major Themes

A total of five main themes were identified as key indicators to inform potential pathological vegetarian or vegan adherence in people with an eating disorder. See Figure SS1 for a flowchart of the themes and their definitions. Before presenting the major themes, we want to acknowledge participant perspectives on the relationship between vegetarianism, veganism, and eating disorders, more broadly. Overall, attitudes toward the interrelationship were heterogeneous. Some participants viewed their history of an eating disorder as being separate from their vegetarianism or veganism. One participant said:

Because of my experience of not liking the taste of meat and going off it, I don't see vegetarian as a predisposer [to developing an eating disorder]. (P9, Pescetarian)

Others emphasized that their vegetarianism or veganism might have been disordered in the beginning but perceived their dietary adherence to evolve into something more genuine as they recovered from their eating disorder and gained clarity.

The first year of treatment, I was like, ‘Oh, is it eating disorder related? I don't know. Everyone's telling me it probably is.…’. But I felt like after a decent amount of treatment and getting to a decent place, even though I did relapse, [veganism] was a separate thing. (P5, Vegan)

Several participants were uncertain whether their dietary adherence was driven by their eating disorder and questioned whether vegetarianism or veganism can healthily coexist with an eating disorder.

I also know that eating disorders tend to find literally any way that they can to manipulate and get away with things. Maybe in the future they can be vegan in a healthy way, but at that point in time, they probably just can't. (P3, Vegetarian)

Despite the overall diverse perspectives detailed above, consensus was reached on the impacts of adhering to veganism for eating disorder recovery. Participants noted that maintaining veganism in recovery was difficult and many participants transitioned to vegetarianism to assist in recovery. One participant described:

In my experience, the post‐binge guilt and shame was amplified significantly and driven a lot by my desire to be vegan. It was only when I changed my diet to nonvegan that I was able to get out of the binge‐purge‐restrict cycle, which meant for me that veganism was driving and getting in the way of my recovery. (P12, Vegan)

#### 3.2.1 Theme 1. Timing Matters

This theme captures the timing of the onset of eating disorder symptoms and the uptake of vegetarianism and veganism, which may provide insights into potential pathological dietary adherence. Participants noted that if eating disorder symptom onset and vegetarian or vegan uptake are closely aligned, this may be a cause for concern.

Asking about the timeline when they went vegetarian or vegan, and then when the eating disorder started, I think that's really important. (P8, Vegetarian)

I think that if you're vegetarian or vegan prior to getting your eating disorder, then being vegetarian and vegan whilst [in] treatment is not an issue. (P7, Semi‐Vegetarian)

Some participants reported that transitioning to vegetarianism or veganism while experiencing an eating disorder worked to maintain their symptoms. One participant noted that their veganism ramped up their eating disorder symptoms:

I guess veganism and additional food rules was the catalyst for my eating disorder to really ramp up. (P9, Pescetarian)

Participants spoke to a specific catalyst or historical event that prompted their vegetarian or vegan adherence. Such event was described as being a potential indicator of genuine adherence. For example, some participants spoke to a traumatic life experience or childhood memory involving meat or the slaughter of animals, with this later prompting their vegetarian or vegan uptake. One participant commented:

It's really easy to say, ‘animal welfare’, but if you were to go a little bit further and say, ‘Was there something in particular that you saw?’, ‘Did you speak to someone in particular?’ Because if they can't tell you specifically what it was [that motivated them to be vegetarian/vegan], chances are that it's a choice that's coming from somewhere else [e.g., a disordered place]. (P11, Vegan)

#### 3.2.2 Theme 2. Explore Motivations for Dietary Adherence

This theme reflects motivations for adherence and maintenance of vegetarianism or veganism as being important indicators for potential pathological adherence. There was a common theme among participants that motivations that are value‐ or belief‐driven, such as animal welfare, environmental concern, or family tradition or culture, were supportive of genuine dietary adherence.

So originally, I think that not wanting to eat animals was from an ethical place. I don't think any sort of eating disorder had developed back then. (P5, Vegan)

Understanding about family dynamics is really [important], and meals as a child and stuff like that is a really interesting way to see how someone has put pieces together as to how they might have chosen to become vegetarian. (P13, Vegetarian)

On the other hand, it was noted that health‐related motivations may be a “red flag” or a cause for concern indicating potential pathological vegetarian or vegan adherence. One participant said:

[Someone saying], ‘Oh, I'm vegan or vegetarian for these specific reasons’—they may not have that indication, or it could be on the other hand that it's very well practiced, in terms of, no, I'm doing it for this, this, this and this. And it might be that they're saying, these are the health benefits. (P4, Vegetarian)

Health‐related motivations were often used synonymously by participants to include weight loss or weight restriction reasons. Some participants motivated by “health” noted that the uptake of their dietary adherence was tied in with looking a specific way, being thin, or avoiding eating.

I was very motivated by weight loss and a fear of weight gain. I had a perception that a vegan diet correlated with a low weight. In my experience I often used the excuse of, ‘I can't eat that because I'm vegan’ as a form of control and restriction; a way to avoid food. (P12, Vegan)

I really don't equate weight loss with vegetarianism—truly in my brain it's not a thing. But I do with veganism because of dairy and it's in a lot of cakes and chocolates. (P9, Pescetarian)

A smaller number of participants described their vegetarian or vegan adherence was driven by taste, texture, or aroma aversions. This may be particularly prevalent in people who have been vegetarian or vegan since childhood or with a family history of vegetarianism or veganism (e.g., parents or siblings). This motivation was generally considered by participants to indicate genuine adherence, particularly it was accompanied by long‐term adherence.

I also was not finding it enjoyable to eat [meat] and the taste and everything just kind of didn't sit right with me. (P2, Vegetarian)

#### 3.2.3 Theme 3. Fear Reaction Cause for Concern

This theme captures a fear‐based reaction to eating meat and/or animal products which may indicate potential pathological vegetarian or vegan adherence. Participants noted that individuals with a healthy relationship with food and their bodies may react to accidentally consuming meat and/or animal products with annoyance, disgust, sadness, or disappointment.

When you get the wrong meal at a restaurant, you're like, ‘This is awkward, but it's not the end of the world’. (P12, Vegan)

On the other hand, in individuals whose dietary adherence may be driven by their eating disorder, accidentally consuming meat and/or animal products might produce a fear‐based response. One participant commented:

I think the [response to being served nonvegan or vegetarian food] would just be very much panic and fear, as opposed to like, sadness and disappointment. It would be quite acute, and it would be very high emotions. (P7, Semi‐Vegetarian)

I think there is a level of distress for someone who is a vegan or vegetarian in recovery, who might feel uncomfortable if they accidentally got something that wasn't vegan. There's a lot more emotion and maybe fear, shame, guilt and all these other things that are not normal vegan sort of behaviors. (P12, Vegan)

Some participants noted that it may be difficult to differentiate these reactions to infer genuine versus pathological adherence. However, exploring drivers behind the reaction (i.e., fear of calories, guilt or grief that an animal has died for the meal, anger toward people who continue to consume meat) may provide insights. For instance, one participant noted:

I feel if someone's reasoning behind being vegetarian or vegan is because of that restrictive factor, I feel like it could go either way. Like either being upset because it's not a safe food for them or on the other hand it could be that they're not even bothered by it because the reasoning behind it isn't about the animal welfare side, it's more of a restrictive thing. (P8, Vegetarian)

#### 3.2.4 Theme 4. Flexibility Within Vegetarianism or Veganism

This theme captures that additional restrictions within the realms of a vegetarian or vegan diet may be an indicator of pathological adherence. For example, if a client has imposed additional restrictions on the foods they are willing to consume (e.g., processed foods, gluten free, whole foods), despite the foods themselves being vegetarian or vegan, or when vegetarian or vegan alternatives are available.

Ultimately, in my eating disorder, I was more scared of processed foods, and you can get vegan foods that are processed. Then I found out about the wholefood plant‐based diet. That was a negative turning point in the reactivation of my eating disorder. (P2, Vegetarian)

If there are restrictions within the restrictions, that's when you can probably get concerned. (P11, Vegan)

Someone whose vegetarianism or veganism may not be tied to their eating disorder may be flexible in their eating practices. For example, they may consume a wide range of foods within those available to them as a vegetarian or vegan. For instance, participant 11 goes on to say:

For me, when my eating disorder takes over, the list of foods that I will eat becomes ridiculously small… The portion sizes shrink, the food variety shrinks, and it's clearly identifiable to me what the difference is because if I'm choosing to not eat, like oats anymore, that's not a vegan choice. That's an eating disorder choice. (Vegan)

Participants noted being open and proactive in seeking plant‐based alternatives during treatment in an effort to remain vegetarian or vegan (i.e., mock meats, dairy alternatives) may be an indicator of genuine dietary adherence.

If you're in recovery, and you're adamant to stay vegan or vegetarian, you would do your own research. You would look into the fact that there's all these alternatives out there, or ‘I can have beans instead of this’. (P6, Vegetarian)

#### 3.2.5 Theme 5. Hold Space for Eating Disorder Deception

This theme reflects participant sentiments that eating disorders may cause diminished insight, particularly around whether one's vegetarian or vegan adherence may be driven by their eating disorder. Participants noted that this relationship can often be difficult to unpack, due to the deceptive nature of eating disorders.

Eating disorders can be sneaky and people can sort of not be quite honest with themselves and their team about their reasoning behind their plant‐based diet. (P1, Vegan)

For a long time, I didn't believe that I had an eating disorder at all. I was like, ‘No, I'm eating healthy. I just eat normally. It couldn't be me. I love food too much’. And I think that also comes back to that when you are suffering with an eating disorder, you don't necessarily know. So, it's really, really, really difficult. (P3, Vegetarian)

Throughout the treatment journey, individuals may gain additional insight into their underlying motivations for vegetarian or vegan adherence, and how it may be linked to their eating disorder. One participant commented:

At the time, I sort of perceived it as something that was ethical…but throughout treatment, I realized that it was kind of a security blanket for my eating disorder. It exacerbated the feelings of shame and guilt when I ate something that wasn't vegan, which did happen at times, and it just made what was already challenging, really a lot worse. (P12, Vegan)

## 4 Discussion

This study aimed to integrate lived experience perspectives to identify and conceptualize indicators of potential pathological vegetarian or vegan adherence in people with an eating disorder. Being able to potentially quantify genuine vegetarian or vegan adherence from eating disorder‐driven behaviors and attitudes may have clinical relevance in terms of modified treatment processes and maintaining autonomy in clients. We identified five themes that participants described as important factors to consider when exploring potentially pathological dietary adherence in people with an eating disorder: (1) *Timing matters*, which captures the timing of the onset of eating disorder symptoms and the uptake of vegetarianism and veganism, (2) *Explore motivations for dietary adherence*, which reflects the motivations for adherence and maintenance of vegetarianism or veganism, (3) *Fear reaction cause for concern*, which captures a fear‐based reaction to eating meat and/or animal products, (4) *Flexibility within vegetarianism or veganism*, which considers additional restrictions within the realms of a vegetarian or vegan diet, and (5) *Hold space for eating disorder deception*, which reflects participant sentiments that eating disorders may cause diminished insight.

Findings demonstrated that the timing of dietary adherence and the onset of eating disorder symptoms may be an important consideration for exploring potentially pathological vegetarian or vegan adherence. Indeed, this theme supports anecdotal reports that inpatient services may require that a client has been vegetarian or vegan for a set period (e.g., 2 years) to be eligible to continue their dietary adherence in treatment (McLean et al. 2025). Recent qualitative findings using the same sample found participants frequently needed to justify their dietary adherence to health professionals, and this was commonly done through food logs, evidence of weight restoration, or timelines of dietary uptake versus eating disorder symptoms (McLean et al. 2025). Most participants in our study confirmed that eating disorder symptoms preceded the uptake of vegetarianism or veganism, which was a cause for concern. This may be particularly relevant as transitioning to a vegetarian or vegan diet requires substantial planning, forethought, and education of food and nutrition (Santini et al. 2025). For example, the process of reading the food labels at the supermarket may elevate symptoms in people with an eating disorder (Putra et al. 2023), compared to longer term vegetarians or vegans whose choices at the supermarket may be more automated as they are already aware of suitable plant‐based products. Participants indicated a specific event marking the uptake of a vegetarian or vegan diet could potentially help distinguish genuine adherence. Such catalyst events were commonly linked to animal welfare (i.e., seeing an animal being harmed) or environmental concerns (i.e., watching a documentary on the impact of livestock‐related emissions) and evoked strong emotion. Emotive catalyst events support the likelihood of genuine dietary adherence and may be unlikely to be linked to health, weight loss, weight restriction, or body image concern.

Motivations were shown to be a potentially important aspect of exploring pathological dietary adherence in vegetarian and vegan clients (Curtis and Comer 2006; Sieke et al. 2022). This finding has been long explored within the literature (Heiss et al. 2017), with partial support in vegetarians but not vegans (McLean et al. 2022b). Among vegetarian samples, individuals who were motivated by weight concerns were found to report significantly higher dietary restraint scores compared to ethical‐, religious‐, or taste‐motivated individuals. On the other hand, literature has demonstrated no differences across adherence motivations among vegans (McLean et al. 2022b). There was a common thread in our findings that motivations that were value‐ or beliefs‐driven, such as animal welfare concerns or religious beliefs, may provide a distinguishing factor to untie eating disorder symptoms. Indeed, this may also be linked with a catalyst as mentioned above. Value‐ or beliefs‐driven motivations were generally considered by participants as more legitimate motivators compared to individuals who were vegetarian or vegan for health or body‐image concerns. It may be useful for health professionals to explore their clients' definition of “health”, as this was noted by participants in our study to be frequently used synonymously for weight loss or weight restriction means and may tie into the broad misassumption that vegetarian and vegan foods are “healthier” (Huang et al. 2016).

A fear‐based reaction to the consumption of meat and/or animal products was noted to be a cause for concern, potentially indicating a disordered relationship with these foods. This could also include an emotional reaction that is out of proportion to the circumstances. Participants noted that the average vegetarian or vegan with a healthy relationship with food and their body may experience frustration, grief for the animal, or disappointment when faced with the consumption of meat and/or animal products, rather than fear. From a health professional perspective, it may be useful to explore the driving force behind the fear reaction and whether it is associated with a fear of calories or weight gain. This generated theme also aligns with Item 5 (“The thought of accidentally eating meat causes you significant distress”) of the V‐EDS and could be used to start a conversation with a client about their aversion to the consumption of meat (McLean et al. 2024a).

Participants noted that maintaining flexible eating habits—where a variety of foods are consumed in a balanced approach—within a vegetarian or vegan context is important. Participants emphasized that flexibility within the context of a vegetarian and vegan diet may indicate that an individual has a healthy relationship with food and their body. On the other hand, someone who places additional restrictions on the foods available to them within the realms of a vegetarian or vegan diet may indicate disordered eating practices, which may align with orthorexia‐type symptoms (Cena et al. 2019). To explore this theme in clinical practice, health professionals may consider using Item 3 (“*A balanced diet can include eating processed plant‐based products [e.g., mock meats]*”) of the V‐EDS to query a client's rigidity around the consumption of processed plant‐based products. This item may also provide insight into “clean eating” practices commonly associated with vegetarianism and veganism (McLean et al. 2024a). Indeed, the consumption of processed plant‐based products may be particularly relevant for vegetarians and vegans seeking eating disorder recovery and where weight restoration is required. While the V‐EDS provides a promising tool to query these themes, current research is preliminary in nature and further research is needed to validate the items, including across a range of eating disorder diagnoses.

While the themes of (1) *Timing matters*, (2) *Explore motivations for dietary adherence*, (3) *Fear reaction cause for concern*, and (4) *Flexibility within vegetarianism or veganism* may be appropriate indicators of pathological dietary adherence in vegetarians and vegans, participants also recognized that health professionals may experience challenges when assessing this information due to the nature of eating disorders. Theme (5) *Hold space for eating disorder deception* highlights and acknowledges this consideration. Participants also acknowledge that there is a degree of nondisclosure for some people experiencing eating disorders and this may impact personal insights into their relationship with their vegetarian or vegan diet. This may also be difficult to assess from a health professional perspective. However, additional insights into a client's own eating practices are likely to grow as recovery progresses.

## 5 Limitations

A limitation of the present study is that participants were primarily female‐identifying, therefore limiting the generalizability of the findings to males and gender‐diverse groups. Despite our best efforts to recruit participants of various genders, no men responded and participated in the study. Next, inclusion criteria required that participants were living in Australia and therefore this study encapsulates the cultural makeup of this country. Information regarding race and religious practices was not collected, thus presenting a limitation to our study. This is particularly important when considering the cultural perceptions held toward meat‐eating around the world. For example, some Eastern countries are noted to have more open attitudes toward vegetarian and vegan food products, which may impact how vegetarian and vegan adherence is approached in the context of eating disorder treatment (Rothgerber and Rosenfeld 2021; Ruby 2012).

## 6 Future Research

Much work is needed to improve our understanding of vegetarian and vegan diets and eating disorder psychopathology. With respect to the findings at hand, further work is needed to validate our generated themes through quantitative research. Specifically, a large sample study would be useful to make comparisons across diagnoses, healthcare professions, and treatment services (i.e., inpatient, outpatient). Future research should continue to explore how vegetarianism or veganism may interact with eating disorder symptoms across a range of demographics. Gender may be particularly pertinent given the varying presentation and prevalence of eating disorders and disordered eating symptoms in men and women, as well as gender‐diverse individuals (Murray et al. 2017; Nagata et al. 2020). More broadly, there is a clear focus on cross‐sectional study designs within the vegetarian and vegan eating disorder field. Only one study has longitudinally explored symptom severity in eating disorder patients across dietary adherences using retrospective clinical chart review, finding no differences in outcomes (Heiss et al. 2021). The potential causal or bidirectional relationship between vegetarian and vegan diets and eating disorders remains unclear, and further longitudinal research is very much needed.

## 7 Clinical Implications

The findings generated from this study may be useful for consideration into clinical practice. We encourage health professionals to spend time understanding their client's vegetarianism or veganism in treatment. This may be best suited within the first few sessions to provide a baseline assessment and allow the health professional to understand the parameters of their client's dietary adherence. Indeed, our generated themes could be useful to start this conversation (McLean et al. 2024a). For example, exploring flexibility within vegetarianism or veganism but also the consumption of meat and/or animal products could provide health professionals with insights into their clients' openness to the consumption of such products which may be required as part of eating disorder recovery. Undoubtedly, this conversation should be had in an open and nonjudgmental manner. It may also be important to continue these discussions throughout treatment as the client may gain additional insight as treatment progresses.

## 8 Conclusions

This study provides insights into untying genuine vegetarian or vegan adherence from potentially pathological adherence in people with an eating disorder. We recruited 17 participants to qualitatively describe their lived experiences of seeking eating disorder treatment while adhering to a vegetarian or vegan diet. Our findings demonstrated several key indicators for further exploration in clinical practice when working with these groups, namely, timing, motivations, fear reaction, and flexibility. We also acknowledge, alongside the participants, that eating disorders may be associated with diminished insight, particularly around driving or maintaining mechanisms where vegetarianism or veganism may play a role. Being able to potentially quantify genuine vegetarian or vegan adherence from eating disorder‐driven behaviors and attitudes provides a valuable stepping stone for the future development of clinical guidelines for the treatment of these groups. Future research is very much needed in this area to explore how to integrate vegetarianism and veganism into eating disorder care where appropriate and desired. This should be done by integrating health professional and loved experience perspectives (Phillipou et al. 2025).

## Author Contributions

**Courtney P. McLean:** conceptualization, methodology, formal analysis, data curation, writing – review and editing, writing – original draft, project administration, funding acquisition. **Kathleen de Boer:** formal analysis, data curation, writing – review and editing. **Megan Bray:** data curation, writing – review and editing.

## Conflicts of Interest

The authors declare no conflicts of interest.

## Supporting information


Data S1.

Figure S1.

## Data Availability

The data that support the findings of this study are available on request from the corresponding author. The data are not publicly available due to privacy or ethical restrictions.
