# Supplementary figures and images for: “If There Are Restrictions Within the Restrictions, That's When You Can Probably Get Concerned”: Key Indicators for Untying Vegetarianism and Veganism From Eating Disorder Pathology
Source: Int J Eat Disord. 2025 May 23;58(9):1723–31. doi: 10.1002/eat.24475 (PMC12423574; doi:10.1002/eat.24475)

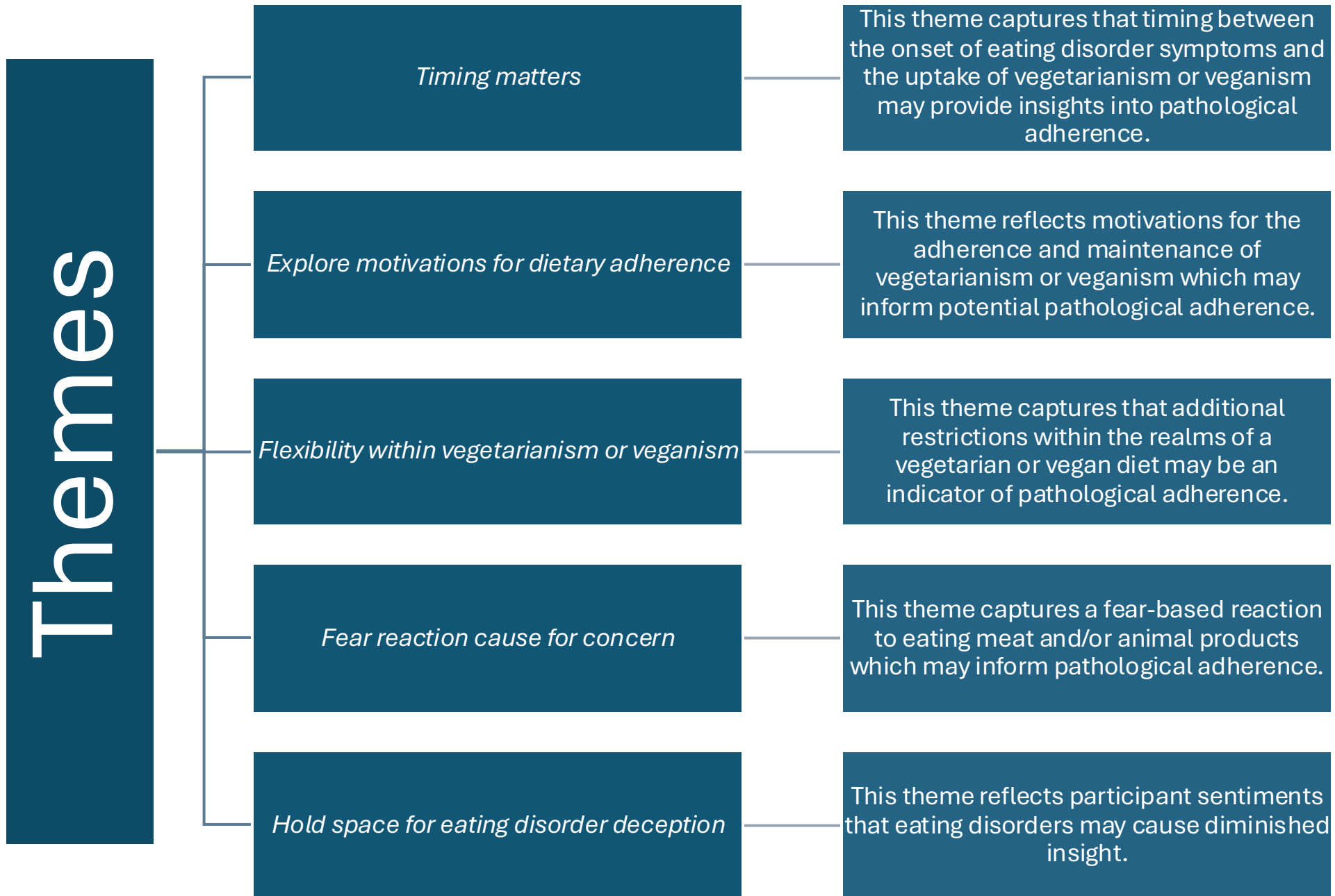

**Figure 1.** Flowchart of the major themes and their definitions.

Supplement: Supplementary file 2 — Figure S1. [file EAT-58-1723-s002.pdf]
